# Novel *VAC14* Variants Identified in a Patient with Striatonigral Degeneration and Prolonged Survival

**DOI:** 10.1002/mdc3.70152

**Published:** 2025-05-30

**Authors:** Silvestre Cuinat, Christèle Dubourg, Gaël Nicolas, Jean‐Madeleine de Sainte Agathe, Sylvie Odent, Laurent Pasquier, Audrey Riou

**Affiliations:** ^1^ Service de Génétique Clinique CRMR anomalies du développement CLAD‐Ouest, CRDI (Centre de Référence Déficiences Intellectuelles de Causes Rares), CHU Rennes Rennes France; ^2^ Laboratoire de Génétique Moléculaire Hôpital Pontchaillou, CHU Rennes Rennes France; ^3^ CNRS, INSERM UMR 6290, ERL U1305, F‐35000, Université de Rennes, IGDR Rennes France; ^4^ Department of Genetics Univ Rouen Normandie, Normandie Univ, Inserm U1245 and CHU Rouen Rouen France; ^5^ LBM SeqOIA Paris France; ^6^ Département de Génétique Médicale Hôpital Pitié‐Salpêtrière, AP‐HP, Sorbonne Université Paris France; ^7^ FHU GenOMedS, ERN ITHACA, CHU Rennes Rennes France; ^8^ Service de Neurologie CHU Rennes Rennes France

**Keywords:** dystonia, neurodegeneration with brain iron accumulation, spastic tetraplegia, striatonigral degeneration, *VAC14*

Neurodegeneration with Brain Iron Accumulation (NBIA, [MIM:PS234200]) are Mendelian disorders, associated with excessive iron deposition in the brain, particularly in globi pallidi, with extrapyramidal, pyramidal, cognitive, psychiatric symptoms, and early death. To date, >10 genes are associated with NBIA.

Recently, biallelic pathogenic variants in *VAC14* were reported in children with striatonigral degeneration^1^ and NBIA.^2^ Since then, 17 patients from 13 different families have been identified.^1^, ^2^, ^3^, ^4^, ^5^, ^6^, ^7^, ^8^

We report here a 37‐year‐old patient with rapid striatonigral degeneration, which started at the age of 2 years, followed by a clinical stabilization, while his sister died at 20 from the same disease. We identified two novel variants in *VAC14* by trio‐based genome sequencing.

A 37‐year‐old (y.o) male, second child born from unrelated parents with unremarkable family history, had a normal initial neurodevelopment. He presented with ambulation difficulties from age 2, walking on his toes, then quickly lost his ability to walk and speak. At 14 y.o, he suffered from severe motor disability requiring an electric wheelchair. Neurological examination showed tetraspasticity, extrapyramidal hypertonia, generalized dystonia, but preserved cognition. Dysphagia required a gastrostomy tube. Increased auditory evoked potentials suggested brainstem involvement. At 37, his condition appeared to have been stable for several years (Fig. 1A–C). He could read and communicate through a computer. He developed vertical gaze palsy. At 14 y.o, brain MRI suggested bilateral abnormalities of medial globi pallidi (GPm) and striatum. At 20 and 33 y.o, these abnormalities were evident, with cerebral peduncles and putamen T2‐FLAIR hyperintensities, GPm T2‐FLAIR hypointensities, and pallidal T_2_* hypointensity suggesting metal deposits. The ventricles were slightly enlarged, associated with cortico‐subcortical, cerebellar and putaminal atrophy, with a slight evolution over the past 17 years (Fig. 1D–M).

**Figure 1:**
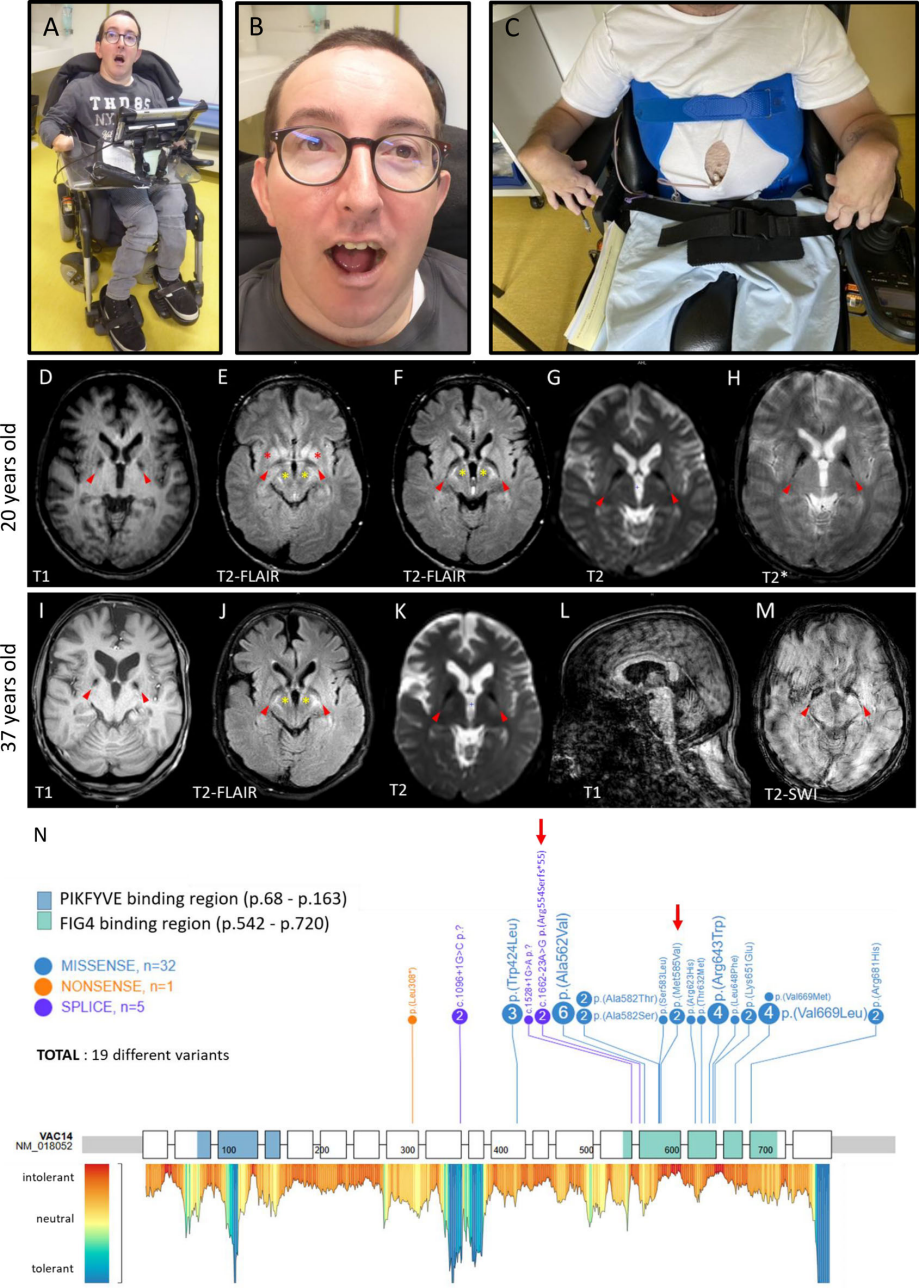
Clinical and radiological features of the proband. (A–C) Pictures of the proband at 37 years of age. Note orofacial dystonia resulting in a chronic subluxation of the jaw (A, B), high forehead, bitemporal narrowing, thin upper lip, hypoplastic nasal wings, slightly low‐set ears (B), severe hyperflexion of the wrists, mallet fingers (C). (D–M) Brain MRI of the patient at the age of 20 (D‐H) and 37 years (I–M), showing GPm “comet tail” hyposignal on T2‐FLAIR (red arrows; E, F, J), T2 (red arrows; G, K), T_2_* (red arrows; H), T1 sequences (red arrows; D, I), and T2‐SWI (M), hyperintensities of putamen on T2‐FLAIR sequences (red and yellow stars; E, F, J), T2‐FLAIR hyperintensities of cerebral peduncles and posterior limbs of internal capsules suggesting pyramidal damage (yellow stars; E, F, J), striatum atrophy on T1 sequence (D, I), evolutive cortico‐subcortical atrophy, particularly in the frontal region, with ventricular enlargement (D, I), and cerebellar atrophy (L). Note the stability of the lesions over 17 years, apart from the progression of the cortico‐subcortical atrophy. (N) Genetic findings of individuals with *VAC14* variants. A schematic view of *VAC14* exon sequence (NM_16120.3), its Metadome missense tolerance landscape (https://stuart.radboudumc.nl/metadome/) and the variants identified in the literature. The amino acid numbering and the protein domains are based on PeCan Data of Proteinpaint. The red arrows indicate the variants identified in the family described here.

His sister had a similar history. After an initially normal neurodevelopment, she also presented with progressive spastic paraparesis, followed by language regression. At the age of 7, she lost her ability to speak. Her intellectual functions were preserved. She died at 20, following inhalation pneumopathy.

After a negative exome sequencing, genome sequencing was performed on the proband and his unaffected parents (Supplemental Methods). Two *VAC14* (NM_018052.5) variants in compound heterozygous state were identified: chr16(GRCh38):70698720T>C c.1753A>G p.(Met585Val) inherited from the father, and chr16(GRCh38):70698834T>C c.1662‐23A>G p.? inherited from the mother, both rare in general population (1 and 0 individuals in gnomADv4.1.0, respectively). The p.(Met585Val) missense variant was predicted as deleterious (REVEL = 0.48; CADD = 23). The intronic variant c.1662‐23A>G was predicted to disrupt the nearby 3′splice site of intron 14 (SpliceAI), as confirmed by a *VAC14* transcript analysis: RT‐PCR revealed an intronic retention of the last 143‐nucleotides of intron 14 (r.1662‐143_1662‐1) resulting in a frameshift p.Arg554Serfs*55 and supporting a loss‐of‐function effect. Both variants have therefore been reclassified as likely pathogenic.

Thus, the two siblings had a *VAC14*‐related neurodegeneration manifested by early motor and language regression with progressive spastic tetraparesis, and absence of intellectual impairment. While the elder sister died at 20 y.o, the proband aged 37 is the oldest patient reported to date, with a noticeable stabilization of the disease over several years, and an MRI pattern suggestive of NBIA.

*VAC14* biallelic pathogenic variants are now reported in 19 patients from 14 different families. After a normal neurodevelopment, motor and language regression occurs before 5 y.o in 70% of cases.^1^, ^5^ Later‐onset forms manifest as progressive generalized dystonia in childhood or adolescence, with or without spasticity.^2^, ^4^, ^6^, ^7^, ^8^ Over the disease progression, motor disability worsens, while intellectual capacities are preserved in most cases (16/19). Clinical and radiological features are summarized in Tables S1 and S2.

We noted singular elements in our patients’ history: they presented a very rapid motor and language deterioration, but while most patients enter the disease with dystonia, both showed pyramidal syndrome in the foreground, evoking hereditary spastic paraplegia. A vertical gaze ophtalmoplegia was lately observed in the proband, possibly due to the mesencephalic involvement as reported previously.^6^ A moderate frontal cortico‐subcortical atrophy was observed, not reported before, perhaps due to the lack of neuroradiological descriptions in adult patients. The prolonged survival and clinical stability of the proband is also remarkable, since only 3/17 patients have been reported in adulthood.^4^, ^5^, ^6^

Despite a typical NBIA pattern, the symptoms could be better explained by neuronal death and cavitation, as suggested by T2‐FLAIR hyperintensities of putamen and cerebral peduncles. Indeed, the ferromagnetic artifact is observed in 37% patients (7/19), who appear older (mean age 16 y.o [range: 9–29]) and with a longer disease course (mean 7 years [0–18]), compared with those presenting only a lesional pattern in basal ganglia (mean age 2.1 y.o [1–3]; mean duration 0.8 years [0–2]). This suggests that neuronal cell death may precedes iron accumulation (Table S2), as supported by neuropathology of young siblings showing neurodegeneration with vacuolization, but no iron deposition,^9^ and *Vac14*‐hypomorphic mouse model showing extensive spongiform neurodegeneration.^10^ However, the clinical improvement after GPi‐DBS in one patient with *VAC14*‐NBIA^2^ would also suggest a neuronal dysfunction, rather than a pure, irreversible lesional process.

A total of 19 different variants are now identified, mostly missense changes aggregating in the VAC14 dimerization domain (Fig. 1N). Genotype–phenotype correlation is difficult to establish, but the only patient with congenital presentation carried the only nonsense variant reported.^3^ Complete *VAC14* biallelic loss‐of‐function has never been reported in humans, suggesting a lethality of this condition as reported in mice,^10^ and raising the hypothesis of a correlation between clinical severity and residual VAC14 amount.

In conclusion, we describe siblings with *VAC14*‐related disorder, carrying two novel pathogenic variants. The proband, oldest patient reported so far, had a 35‐year history of disease progression, with surprising clinical stabilization, prolonged survival and no cognitive impairment, bringing a novel perspective for prognosis.

## Author Roles

(1) Research project: A. Conception, B. Organization, C. Execution; (2) Statistical analysis: A. Design, B. Execution, C. Review and critique; (3) Manuscript preparation: A. Writing of the first draft, B. Review and critique.

S.C.: 1A, 1B, 1C, 2A, 2B, 3A.

C.D.: 1A, 1B, 1C, 2C, 3B.

G.N.: 1A, 1B, 1C, 2C, 3B.

J.M.S.A.: 1A, 1B, 1C, 2C, 3B.

S.O.: 1B, 1C, 2C, 3B.

L.P.: 1A, 1B, 1C, 2C, 3B.

A.R.: 1A, 1B, 1C, 2C, 3A, 3B.

## Disclosures

**Ethical Compliance Statement:** Genetic analyses were subject to informed consent by the families following a specialized genetic consultation in Rennes UMC, in accordance with French guidelines, bioethics laws and the General Data Protection Regulation (RGPD), framed by the National Commission for Information Technology and Civil Liberties (CNIL). A specific consent for the publication of the patient's photographs was obtained. We confirm that we have read the Journal's position on issues involved in ethical publication and affirm that this work is consistent with those guidelines.

**Funding Sources and Conflicts of Interest:** The authors have no funding nor conflicts of interest to declare in the context of this work.

**Financial Disclosures for the Previous 12 Months:** All authors declare no financial or non‐financial competing interests.

## Supporting information


**Data S1** Supplemental methods: Detailed method of genetic investigation in the proband and his parents.

**Table S1** Summary of clinical and neuroradiological characteristics in patients with *VAC14*‐related disorders, in this study and in literature

**Table S2.** Detailed clinical and neuroradiological characteristics in patients with *VAC14*‐related disorders, in this study and in literature

## Data Availability

The data that support the findings of this study are in the supplemental data section.
