# Supplementary material for: Novel VAC14 Variants Identified in a Patient with Striatonigral Degeneration and Prolonged Survival
Source: Mov Disord Clin Pract. 2025 May 30;12(10):1668–71. doi: 10.1002/mdc3.70152 (PMC12528970; doi:10.1002/mdc3.70152)
Supplement: Supplementary file 1 — Data S1 Supplemental methods: Detailed method of genetic investigation in the proband and his parents. [file MDC3-12-1668-s003.docx]

**SUPPLEMENTAL METHODS**

**Detailed method of genetic investigation in the proband and his parents**

Whole genome sequencing was performed by the Seqoia-FMG2025 French platform as following: DNA was extracted from patient and parental blood samples (DNA from the affected sister was unavailable). Nucleic acid quantification and qualification were performed on Spark and Fragment Analyzer respectively. Fragments were generated by sonication (LE220plus). Size selection and subsequent purification steps were performed on magnetic beads (Sera-Mag magnetic beads). The libraries were quantified by qPCR, and pair-end sequenced (2 x 150 cycles) using SBS technology (Flow Cell S4, NovaSeq 6000). Sequenced files (.BCL) were demultiplexed (bcl2fastq, v2.20.0.422). Alignment of the resulting files (.FASTQ) to the reference genome (GRCh38) used a Burrows-Wheeler transform (BWA-MEM, 0.7.15). Database quality score recalibration was performed by GATK4 (v4.1.6.0, Broad Institute). SNVs and indels were called by GATK4 (v4.1.7.0, Broad Institute) and annotated using SNPeff (4.3t) and SnpSift (4.3t). The databases queried were as follows: SNPEff (v4.3t), 1000Genomes (v2013-05-02), gnomAD (v3), ClinVar (v20190722), COSMIC (v89), dbscSNV (v1.1), dbSNP (v20180418), dbNSFP (v4.0), phastCons (v08) and spliceAI. CNVs were called by WiseCondor v1.2.1 and ClinSV v1.2.1 and annotation was performed using AnnotSV (v2.5.1). Pathogenicity of the *VAC14* variants was established according to American College of Medical Genetics and Genomics/Association for Molecular Pathology (ACMG/AMP) criteria (13). *VAC14* splicing study was performed from RNA extracted from blood samples on PAXgene tubes (RNA Blood Nucleospin®). *Vac14* transcripts were amplified by RT-PCR, visualized on agarose gel, then analyzed after Sanger sequencing. Primer sequences are available upon request.
